# Outcome of low dose cyclophosphamide for induction phase treatment of lupus nephritis, a single center study

**DOI:** 10.1186/s12882-016-0361-0

**Published:** 2016-10-07

**Authors:** Mahesh R. Sigdel, Mukunda P. Kafle, Dibya Singh Shah

**Affiliations:** Department of Nephrology, Tribhuvan University Teaching Hospital, Kathmandu, Nepal

**Keywords:** Induction phase treatment, Intravenous cyclophosphamide, Lupus nephritis, Remission

## Abstract

**Background:**

The current standard for induction phase treatment of lupus nephritis is steroid combined with mycophenolate mofetil or pulse intravenous cyclophosphamide (IVC). The lowest dose of IVC recommended for induction therapy is that used in the Euro-Lupus Trial. It is not known whether same cumulative dose of IVC would be effective when given over six months.

**Methods:**

We carried out a prospective, observational study on 41 patients of biopsy-proven lupus nephritis (class III, IV, V or mixed). For induction, patients received six pulses of monthly IVC (500 mg each), along with steroid. Patients were followed up monthly until one month beyond completion of the sixth pulse. The outcomes assessed were complete remission (proteinuria < 200 mg/day or urine albumin nil with serum albumin >35 gm/L, stable estimated glomerular filtration rate (eGFR) if normal at baseline or increase in eGFR by 25 % if abnormal at baseline and normal urinary sediment), response (complete or partial remissions), complications of therapy and death.

**Results:**

Twenty two patients (53.7 %) had class IV nephritis. Eighteen patients (43.9 %) achieved complete remission, 16 (39.0 %) achieved partial remission, yielding an overall response rate of 82.9 %. Nephrotic range proteinuria (UTP ≥ 3 g/day) and severe hypoalbuminemia (serum albumin < 20 g/L) at baseline influenced remission (*p <*0.05). Infection, seen in 12 patients (29.3 %), was the most common complication. Four deaths (9.6 %) were observed, all due to infection.

**Conclusions:**

For induction phase treatment, Nepalese patients with lupus nephritis responded favorably to steroid and low dose IVC of 3 grams given as six monthly pulses.

## Background

Approximately 35 to 50 % patients of systemic lupus erythematosus (SLE) have clinically evident kidney disease at presentation; during follow-up, lupus nephritis (LN) develops in >60 % patients [1–4]. Overall survival in SLE is 92 % at 10 years after diagnosis; presence of LN reduces survival to approximately 88 % at 10 years [2]. LN affects the clinical outcomes both directly by target organ damage and indirectly through complications of therapy [1]. It is a major cause of end stage renal disease (ESRD) and is associated with a greater than four-fold increase in mortality [1, 5].

Use of the International Society of Nephrology/Renal Pathology Society (ISN/RPS) 2003 classification can serve as a guide to initial therapy of LN [2, 6, 7]. The management of active class III, IV or V nephritis consists of an initial or induction phase of aggressive immunosuppression followed by a prolonged phase of less intense therapy [1, 2, 4, 7–9]. The current standard for the initial phase treatment consists of steroid and intravenous cyclophosphamide (IVC) or mycophenolate mofetil (MMF) [1–4, 7, 9–20]. The NIH trials used IVC at 0.5–1 g/m^2^ monthly for 6 months while the Euro-Lupus Nephritis trial used IVC 500 mg every 2 weeks for 3 months, lowering the dose and duration of IVC without sacrificing the efficacy [11, 13].

In our center, lupus nephritis is routinely treated with steroid and IVC at 500 mg monthly pulse for six months. However, no systematic analysis of the outcome has been carried out so far. In this report, we present our experience with 41 Nepalese patients with lupus nephritis and demonstrate that this dosing is clinically effective in our population. We also analyzed the clinical and pathological data, and related these findings to the outcome.

## Methods

### Design and settings

This study was a prospective, observational study conducted at Tribhuvan University Teaching Hospital (TUTH), Kathmandu, Nepal, between 14^th^ April 2011 and 13^th^ April 2013. Ethical approval was obtained from the Institutional Review Board of Institute of Medicine.

### Patients

A total of 41 consecutive patients with lupus nephritis [2, 21] admitted to the nephrology wards of TUTH during the study period were included. Patients were included if they had a kidney biopsy [2, 21], were subsequently started on steroid and monthly pulse IVC for induction treatment and gave informed consent for the study. They were excluded if they did not give consent, had previously received cyclophosphamide or if they chose MMF as opposed to IVC for induction.

Clinical, biochemical and serological information was obtained at baseline and at each return visit. Patients were followed up monthly till one month beyond the completion of the sixth pulse of IVC. For those patients receiving IVC pulses in other hospitals, information was recorded during TUTH visits later. For those patients unable to attend our hospital later, information was recorded telephonically. The patients lost to follow up were considered as non-responders in the outcome analysis. The decision to institute IVC pulses (rather than MMF) in the initial phase of treatment was taken by the treating nephrologists after discussion with the patients and their relatives. Estimated glomerular filtration rate (eGFR) was calculated using Cockroft-Gault formula at baseline and at the final follow up visit [22].

### Protocol

Criteria for performing kidney biopsies in SLE in TUTH included increasing serum creatinine without alternative causes, proteinuria ≥1.0 g/day, proteinuria ≥0.5 g/day plus hematuria (≥5 RBCs/hpf) or proteinuria ≥0.5 g/day plus cellular casts. Renal biopsies were done in real time, USG guided, with assistance of a radiologist, using a Bard biopsy gun. Two cores of renal tissue were taken and the sample was sent to Dr Lal PathLabs, a reference laboratory in India.

Patients in the study received induction phase treatment with steroid and pulse IVC, 500 mg monthly for six pulses. Patients received total of 1000 ml intravenous fluid during CYC infusion, duration of infusion being two hours; none of the patients received mesna. The initial steroid therapy consisted of intravenous methyl prednisolone pulses 500–1000 mg daily for three days, or oral prednisolone 1 mg/kg/day; generally the patients with severe pancytopenia, significant renal impairment, massive proteinuria and active vasculitis were given intravenous methyl prednisolone pulses if they did not have coexisting infections. The dose of prednisolone was brought down to 0.5 mg/kg/day once the first pulse of IVC was given. Patients also received adjuvant therapy with hydroxychloroquine, calcium, proton pump inhibitor (PPI), angiotensin converting enzyme inhibitor (ACEi) or angiotensin receptor blocker (ARB) and prophylactic cotrimoxazole. The dose of prednisolone was tapered gradually; there was no fixed tapering protocol [4, 23, 24].

Complete blood counts were checked close to the 10^th^ day after the first pulse of IVC. Complete blood counts, blood glucose, serum urea, serum creatinine, serum albumin and urine routine and microscopy tests were done every month prior to the next pulse IVC. Proteinuria was quantified by 24 h urinary collection, at base line and then at the completion of third and sixth pulses of IVC. There was no fixed protocol for monitoring serological lupus activity. After the initial phase of treatment, patients were continued on steroid plus any of the three: azathioprine, MMF or three monthly IVC.

### Outcomes

Complete remission (CR) was defined as proteinuria of less than 200 mg/day or urine albumin nil by dipstix with serum albumin >35 g/L, stable eGFR if normal at baseline or increase in eGFR by 25 % if abnormal at baseline and normal urinary sediments (urinary RBC or WBC <5/HPF each) [3, 25, 26].

Partial remission (PR) was defined as proteinuria between 200 mg to 2.9 g/day or fall in proteinuria by at least 50 % with serum albumin at least 30 g/L and stable eGFR if normal at baseline or increase in eGFR by 25 % if abnormal at baseline [3, 25, 26]. Response was defined as either CR or PR.

The adverse outcomes which were documented included complications of therapy, ESRD or death.

### Statistical analysis

For description of baseline characteristics mean ± standard deviation, median with range, frequency and percentages were used. ×^2^ test was used for categorical variables and independent sample *t* test was used to compare the means in different groups. The *p* value of <0.05 was considered statistically significant. All statistical analyses were performed using SPSS software (version 16.0; SPSS Inc, http://www.ibm.com/analytics/us/en/technology/spss/).

## Results

### Patient characteristics

A total of 41 patients were enrolled in the study. Mean age was 26.9 ± 10.6 years (range 14–61 years), female: male ratio was 12.6:1 and the median duration of symptoms was 120 days (range, 4–5110 days). The main presenting symptoms were joint pain (in 70.7 %), limb swelling (in 65.8 %), photosensitivity (in 41.7 %) and fever (in 39.0 %); other symptoms were oliguria, malar rash, oral ulcer and hair loss. Pallor and edema (each present in more than two-thirds) and hypertension (in more than a third) were the three most common examination findings at baseline.

All the patients were positive for antinuclear antibody (ANA), while anti ds-DNA antibody was positive in 87.8 % of them. The majority, 56.1 % had eGFR < 60 ml/min. Anemia, leukopenia and thrombocytopenia were present in 73.2 %, 46.3 % and 26.8 % respectively. Similarly, 87.8 % were hypoalbuminemic (serum albumin < 30 g/L), 41.5 % had severe hypoalbuminemia defined as serum albumin < 20 g/L. Nephrotic range proteinuria was present in 53.7 % patients, mean urinary total protein (UTP) being 3268 ± 2283 mg/day; 58.5 % had microscopic hematuria. Hypothyroidism (clinical and subclinical) was found in 29.3 % patients.

### Renal biopsy findings

**Fig. 1:**
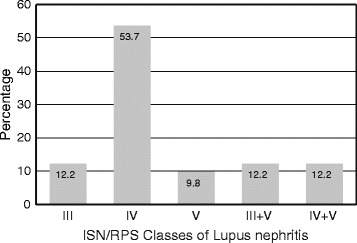
International Society of Nephrology/Renal Pathology Society (ISN/RPS) classes of lupus nephritis in renal biopsy (*n =* 41)

Twenty two patients (53.7 %) had ISN/RPS class IV nephritis (Fig. 1). The mean activity index (AI) was 8.4 ± 4.8 and mean chronicity index (CI) was 3.5 ± 3.2; 16.2 % had combined AI >7 & CI >3.

### Relationship of baseline parameters with classes of lupus nephritis

The patients with class IV LN had a shorter duration of symptoms than other classes (*p =* 0.02); otherwise baseline parameters did not differ significantly between different classes of LN (Table 1). However, class IV patients tended to have higher systolic blood pressure (SBP) and lower platelet count compared to other classes (*p >* 0.05).

**Table 1:** Effects of baseline clinical and investigational parameters on classes of lupus nephrits

Parameter	Class IV	Other classes	*P* value
	(*n =* 22)	(*n =* 19)	
Mean age in years	26.2 (±8.7)	28.7 (±11.5)	0.42
Mean duration of symptoms in days	152.5	785.5	0.02
	(±304.5)	(±1206.2)	
Number of patients with SBP (in mmHg)			
≥140	9 (40.9 %)	5 (26.3 %)	0.33
<140	13 (59.1 %)	14 (73.7 %)	
Mean leukocyte count/mm^3^	5702	5912	0.82
	(±2345)	(±3505)	
Mean platelet count/mm^3^	145,395	206,805	0.09
	(±66092)	(±150947)	
Mean hemoglobin in g%	9.2 (±2.3)	9.1 (±2.4)	0.88
Mean eGFR in ml/min	54.5 (±28)	62.9 (±42.4)	0.45
Number of patients with eGFR			
≥60 ml/min	10 (45.4 %)	8(42.1 %)	0.83
<60 ml/min	12 (54.6 %)	11(57.9 %)	
Mean serum albumin in g/L	21.5 (±5.6)	21.2 (±7.1)	0.88
Mean UTP in mg/day	3903.2	2533.1	0.05
	(±2767.6)	(±126.8)	

*SBP* systolic blood pressure, *eGFR* estimated glomerular filtration rate, *UTP* urinary total protein, (*±*) standard deviation

### Management of patients

The median duration of hospitalization in the first episode was 9 days (range 2-41 days). All the patients received six pulses of IVC, making a total of three grams. Eighteen patients (43.9 %) received intravenous methyl prednisolone (500 mg to 1000 mg) for three days. Fifteen patients (36.6 %) received blood transfusion. All the patients received adjunctive hydroxychloroquine, cotrimoxazole, calcium and oral PPI. A total of 34 patients (82.9 %) were given ACEi or ARB therapy. The mean dose of prednisolone at last follow up was 11.7 ± 4.2 mg/day; 16 patients (42.1 %) were getting prednisolone >10 mg/day while 10 patients (26.3 %) were still on prednisolone ≥ 15 mg/day.

### Outcome

A total of 18 patients (43.9 %) achieved complete remission, 16 (39.02 %) achieved partial remission; there were four deaths and three were lost to follow up. All deaths occurred before achieving remission. The response rate (CR + PR) was 82.9 %. The mean time for urine albumin to become negative in dipstix test was 163.6 ± 44.7 days.

### Relationship of clinicopathological parameters with response

Only nephrotic range proteinuria (urinary total protein, UTP ≥ 3 g/day), severe hypoalbuminemia (serum albumin < 20 g/L) and serum albumin significantly and negatively affected attainment of remission (*p <* 0.05) (Table 2). However, eGFR at presentation did not affect the attainment of CR (*p >* 0.05). Those patients not entering remission were found to have longer duration of symptoms and had higher blood pressure at presentation (*p >* 0.05). The biopsy classes of LN and AI & CI did not influence the remission rate. None of the four dialyzed patients achieved CR (*p >* 0.05).

**Table 2:** Relation of baseline parameters of patients with complete remission (CR) in lupus nephritis

Parameters	CR (*n =* 18)	No CR (*n =* 23)	*P* value
Mean age in years	27.2 (±9.1)	27 (±11.3)	0.97
Mean duration of symptoms in days	438 (±541)	452(±1113)	0.96
Mean SBP in mmHG	124 (±20)	131(±22)	0.28
Mean DBP in mmHG	79 (±12.8)	83 (±12.1)	0.35
Mean eGFR in ml/min	54.5 (±33.9)	61.4 (±36.7)	0.54
Mean serum albumin in g/L	24.8 (±6.3)	18.6 (±4.8)	0.01
Number of patients with serum albumin			
≥ 20 g/L	14 (77.8 %)	8 (34.8 %)	0.006
< 20 g/L	4 (22.2 %)	15 (65.2 %)	
Number of patients with UTP (mg/day)			
≥ 3000	6 (33.3 %)	16 (69.6 %)	0.02
< 3000	12 (66.7 %)	7 (30.4 %)	
Number of patient with AI			
>7	8 (50 %)	13 (61.9 %)	0.67
≤7	8 (50 %)	8 (38.1 %)	
Number of patient with CI			
> 3	4 (25 %)	8(38.1 %)	0.34
≤ 3	12 (75 %)	13 (61.9 %)	
Number of patients with combined			
AI >7 & CI >3	2 (12.5 %)	4 (19 %)	0.54
AI ≤7 & CI ≤ 3	14 (87.5 %)	17 (81 %)	
Number of patients in different classes of LN			
III	4 (80 %)	1 (20 %)	0.29
IV	9 (40.1 %)	13 (59.1 %)	
V	1 (25 %)	3 (75 %)	
III + V	1 (20 %)	4 (80 %)	
IV + V	3 (60 %)	2 (40 %)	
Number of patients			
Dialyzed	0	4 (100 %)	0.06
Not dialyzed	18 (48.6 %)	19 (51.4 %)	

*SBP* systolic blood pressure, *DBP* diastolic blood pressure, *eGFR* estimated glomerular filtration rate, *UTP* urinary total protein, *AI* activity index, *CI* chronicity index, (±) standard deviation

### Adverse events

**Fig. 2:**
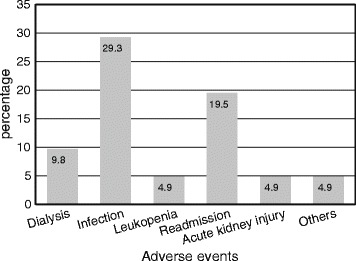
Adverse events encountered during the study (*n =* 41)

Infection was the most common adverse event noted in 12 patients (29.3 %) followed by hospital readmission (19.5 %) (Fig. 2). Pneumonia was the most common infection complicating 10 out of 12 patients, one patient developed urinary tract infection (UTI) and the other one developed herpes zoster. All the four deaths occurred in class IV (*p =* 0.03) and all deaths were due to infection. Acute kidney injury (AKI) resulted from hypovolemia secondary to excessive diuresis in one patient and severe UTI leading to hypotension in the other. When all the adverse events (dialysis, infection, leukopenia, readmission, AKI and others) were combined, the difference between lupus class IV and other classes was not significant (*p =* 0.25).

## Discussion

With the availability of effective treatment options to control aggressive disease in lupus nephritis, the medical community is seeking measures to decrease the toxicity of therapy without compromising efficacy [1, 8, 9, 27]. An ideal therapy for LN would be non-toxic, easy to administer, affordable, would achieve early complete remission, would not require prolonged use, would prevent relapse and essentially cure the disease instead of controlling it. Newer therapeutic options are being explored [5, 28, 29]. Our hospital uses a modified protocol of IVC, discussed before, that is based on local experience, considers the relatively smaller body surface area of our patients and aims to decrease the gonadal toxicity of IVC in young females.

The demographic parameters, symptoms, signs and baseline investigation findings of our cohort in general match with that reported by other workers [3, 6, 25, 26, 30–36]; anemia was more common in our patients, and hypothyroidism was seen in 12 patients (29.3 %). The baseline eGFR and degree of proteinuria is comparable with the renal findings in other studies, but the mean serum albumin was lower than that observed elsewhere [3, 25, 26, 31]. We had class IV nephritis in 53.7 % of our patients, that is comparable with other studies [3, 25, 26, 36]. Our AI and CI indices also match the findings of mean AI score 8.03 ± 5.12 and mean CI score of 2.84 ± 2.2.17 in a study by Yang et al. [37]. These indices were higher than those reported by Chen et al. [26].

In our study, class IV nephritis patients presented earlier than other classes (*p =* 0.02) and tended to have higher systolic blood pressure and lower platelet count (*p >* 0.05); otherwise there was no significant difference between class IV and other classes on any of the other baseline parameters. These findings support the ACR recommendation that all patients with clinical LN, previously untreated, should undergo renal biopsy (unless strongly contraindicated) [2].

Complete remission was achieved in 43.9 % patients in our study; 39.0 % achieved partial remission, 9.6 % died and 7.3 % were lost to follow up. The response rate was 82.9 %. Our response rate was higher than that observed in the ALMS multicenter study, where the response rates were 56.2 % in the MMF group and 53.0 % in the IVC group [3]. In an NIH trial [11], the methylprednisolone and IVC group attained a remission rate of 85 %; in a multicenter Chinese study the CR and response rates to IVC as per NIH protocol were 38.5 % and 82.1 % respectively [26]; these results were very similar to ours. In the Euro-Lupus trial, renal remission was achieved in 71 % of the low-dose group and 54 % of the high-dose group [13]. In the American study by Ginzler et al. [19], response rate to MMF was 52.1 % and in CYC was 30.4 %, rates far lower than what we achieved. In the trial by Chan et al. in 42 Chinese patients, 81 % of the patients in MMF group achieved CR compared with 76 % in the CYC; the response rates were more than 90 % in each [17]. There is one important difference between our study and the other studies. Our patients received a total of 3 grams IVC over six months, while the other studies have used either IVC as per NIH protocol or Euro lupus trial protocol with total of 3 grams of IVC in three months) [11, 13]. The mean dose of prednisolone at last follow up in our study was 11.7 ± 4.2 mg/day, comparable with the mean dose of prednisone on 24^th^ week in the Ginzler study [19]; however, we appreciate that this is still a high dose which could potentially be associated with significant side effects. These findings suggest that our induction regime could be used in Nepalese lupus nephritis patients. This IVC regime exposes the patients of LN to lower cumulative dose of CYC given over longer duration so that the side effects, especially infections and leukopenia will be decreased without sacrificing efficacy.

Our study had good follow up; only three patients (7.3 %) were lost to follow up. In the ALMS induction trial, only 82.7 % patients remained in the study at 24^th^ week [3]. In the Ginzler’s study 17.14 % had to be withdrawn from the study, though only 1.43 % were lost to follow up [19].

Infection and leukopenia have always been major limiting factor in lupus therapy. We had four deaths in our study (9.8 %). All of them were class IV, had significant anemia, hypoalbuminemia and markedly reduced eGFR; they died early in the course due to infection complicating severe disease. This corroborates the observation of other workers that severe disease and infection are two important causes of early deaths in lupus [1, 8, 9]. Chan et al. also reported death rate of 10 %, similar to ours [17]. A lower death rate of 3.78 %, was seen in the ALMS induction study [3]; only three deaths out of 140 patients occurred in Ginzler study [19]. This relatively higher death rate in our study compared to those from the West could be because of a relatively poorer support system and higher ambient infection in Nepal. This was observed in the relatively higher infection rates (29.3 %) without higher rates of leukopenia in our patients. The ALMS study reported an infection rate of 10 % in the IVC group and 12 % in the MMF group [3]. Our infection rate is comparable with that encountered by Chan et al. in their study over a decade ago (19 % in MMF group and 33 % in the IVC group) [17]. Pneumonia was the most common infection observed by us (in 10 out of 12 patients). We feel that balancing immunosuppression in aggressive disease against the risk of potentially fatal infection is the art in the management of lupus nephritis. Other adverse events observed in our study were the need for dialysis (in 9.8 %), readmission (in 19.5 %) and leukopenia, AKI and others in 4.87 % each. We did not observe gastrointestinal side effects, significant alopecia or new onset menstrual irregularities; this is likely because of the lower dose of IVC used.

Multiple factors are known to predict outcome in LN [1, 8, 25, 38, 39]. We found that nephrotic range proteinuria and hypoalbuminemia significantly affected the attainment of remission (*p <* 0.05). Those patients who did not enter remission had higher BP (*p >* 0.05) and none of the dialyzed patients entered CR (*p >* 0.05); age and baseline eGFR did not affect remission rates. These differences could simply reflect the relatively small size of our study population. We could not evaluate the influence of gender, race, anti ds-DNA and complements in outcome. Ayodele et al. showed class IV LN was histological predictor of poor renal outcome [25]. We did not see difference in remission rates when class IV was compared to other classes (*p =* 0.29), however, class IV lupus negatively affected survival in the short term (*p =* 0.03). We could not find the significant influence of activity and chronicity indices in outcomes (*p >* 0.05), unlike the findings of other workers [6, 40]. When all the adverse outcomes were combined, the difference between lupus class IV and other classes was not significant (*p =* 0.25).

The limitations of the present study are that it was an observational single center study with no control group. There was no fixed tapering schedule for prednisolone, some information during follow-up was collected telephonically, and it was a short term study, without long-term follow-up. There are, however, several strengths as well. It is the first prospective study in patients with lupus nephritis in Nepal, with a reasonable sample size and a follow up rate of 92.7 %.

## Conclusions

The response rate of 82.9 %, with complete remission in 43.9 %, suggests that the low-dose protocol used in the study may be useful for initial phase treatment of lupus nephritis without compromising efficacy. However, larger, multicenter, multiethnic, randomised, controlled study is required to provide stronger evidence.
